# Reduced rank regression for mixed predictor and response variables

**DOI:** 10.1111/bmsp.70004

**Published:** 2025-08-28

**Authors:** Mark de Rooij, Lorenza Cotugno, Roberta Siciliano

**Affiliations:** ^1^ Methodology and Statistics Department Leiden University Leiden The Netherlands; ^2^ Department of Physics University of Naples Federico II Naples Italy; ^3^ Department of Electrical Engineering and Information Technology University of Naples Federico II Naples Italy

**Keywords:** generalized linear models, MM algorithm, multivariate regression, optimal scaling

## Abstract

In this paper, we propose the generalized mixed reduced rank regression method, GMR^3^ for short. GMR^3^ is a regression method for a mix of numeric, binary and ordinal response variables. The predictor variables can be a mix of binary, nominal, ordinal and numeric variables. For dealing with the categorical predictors we use optimal scaling. A majorization‐minimization algorithm is derived for maximum likelihood estimation. A series of simulation studies is shown (Section 4) to evaluate the performance of the algorithm with different types of predictor and response variables. In Section 5, we briefly discuss the choices to make when applying the model the empirical data and give suggestions for supporting such choices. In a second simulation study (Section 6), we further study the behaviour of the model and algorithm in different scenarios for the true rank in relation to sample size. In Section 7, we show an application of GMR^3^ using the Eurobarometer Surveys data set of 2023.

## 1 INTRODUCTION

In this paper, we will describe a general methodology for the case where we are interested in the analysis of dependence, that is, we like to see the dependence of a set of variables on another set of variables, as in regression methods. Such an analysis of dependence can be contrasted to an analysis of interdependence (Gifi, 1990), the latter treats the variables in a symmetric way, as in correlation and association analysis.

Usually, researchers have multiple response variables and multiple predictor variables. Typically, researchers analyse the data in a univariate way, one response variable at a time. However, as Fish (1988) argued, it is important to analyse the data using multivariate methods, as they best honour the reality about which the researcher is purportedly trying to generalize. To quote Fish (1988), ‘The reality in which social scientists are interested is usually one in which the researcher cares about multiple outcomes, in which most outcomes have multiple causes and in which most causes have multiple effects’.

With P predictor and R continuous response variables, we could fit a multivariate regression model

$$Y={1m}^{\prime }+\mathrm{XA}+E,$$

that relates the set of response variables with the set of predictor variables. The coefficients of such a regression model when estimated with least squares or maximum likelihood techniques are equal to the coefficients from R separate regression models, one for each response variable. Hence, the fact that the response variables are likely to be related does not play a role in the estimation as no information about the associations is taken into account (Reinsel et al., 2022, chapter 1). A truly multivariate model would take such information into account and model in some way the associations among the response variables. Furthermore, the multivariate regression model estimates many parameters even in simple cases. The matrix A is of size P×R and even with moderate number of predictor and response variables this number is relatively large. Hence, the number of observations needs to be sufficiently large compared to the number of parameters to provide accurate estimates and the interpretation of all regression coefficients can become unmanageable.

Various approaches can be considered to develop a truly multivariate model that (1) takes into account and models the associations among the response variables, (2) reduces the number of parameters and (3) facilitates the interpretation of the final model. The reduced rank regression model also known as redundancy analysis is such an approach. This model was first introduced by Anderson (1951) and further developed by several authors (Davies & Tso, 1982; Izenman, 1975; Tso, 1981; Van den Wollenberg, 1977). An overview of developments for reduced rank models can be found in Reinsel et al. (2022). The key idea is to write the matrix of regression coefficients (A) as a product of two matrices of lower dimensionality, namely the regression weights (B) and the factor loadings (V), that is,

$$A={\mathrm{BV}}^{\prime },$$

where B is a P×S matrix and V is an R×S matrix. The resulting matrix A has rank S whereas the matrix in the multivariate regression model has rank min(P,R), hence the name reduced rank regression. We will use the names rank or dimensionality interchangeable. The user has to choose the required rank or a model selection procedure for choosing an optimal S should be employed. When S=min(P,R) the reduced rank regression model becomes equal to the multivariate regression model. When S<min(P,R) the number of parameters is reduced.

The low rank structure implies that the response variables are dependent on the predictor variables through a small number of latent variables. These latent variables are defined as U=XB, that is, they are linear combinations of the predictor variables. These S latent variables are shared among the responses and as such the reduced rank regression model with reduced S is a truly multivariate model taking into account the associations among the response variables (Luo et al., 2018). When S<min(P,R) the model implies associations among the responses. To see that, take S=1. In that case the model implies associations among *all* response variables: positive associations when the loadings of two response variables have the same sign, negative when the sign differs. When S=2, the model might break up the response variables in two groups, one group with zero loadings on the second dimension, the other group with zero loadings for the first dimension. Variables that pertain to a certain dimension have model implied associations (positive if the sign is equal, negative otherwise). The model implied association for pairs of variables pertaining strictly to different dimensions is null. In data analysis, we usually do not find estimates exactly equal to zero. When S>2, the model becomes more and more flexible in its ability to represent associations. Ultimately, when S=R each response variable might pertain to a certain dimension and the model does not imply the responses to be correlated.

Reduced rank regression can also be considered a constraint principal component analysis (Takane, 2013). In principal component analysis the centred data matrix $Y_{c}=Y-{1m}^{\prime }$ is usually decomposed in a set of object scores and a set of variable loadings, that is,

$$Y_{c}={\mathrm{UV}}^{\prime }+E.$$

Here both the matrices U and V are assumed to have a few (i.e. S) columns. In reduced rank regression, the object scores U of principal component analysis are constrained to be a linear combination of the predictor variables U=XB.

For our exposition in the following sections, we note that the reduced rank regression model for numeric response variables, r=1,…,R can be written as

$$y_{ir}=m_{r}+x_{i}^{\prime }Bv_{r}+\epsilon_{ir},\;\forall \;r$$

where $m_{r}$ is a conditional mean, or intercept for response variable r, $x_{i}$ is the vector with observed values of the predictor variables for observation i, B is a matrix with regression weights, $v_{r}$ is a vector of loadings for response variable r and $\epsilon_{ir}$ is the error component.

Variables can be measured on different scales (Stevens, 1946). It is possible to distinguish between ratio, interval, ordinal and nominal variables. Binary variables can be considered special cases of both ordinal and nominal variables. In a statistical analysis, it is important to take into account the measurement level of the variable, as this defines the kind of operations that are allowed.

In this paper, we consider numeric (i.e. ratio and interval responses), binary and ordinal response variables. These types of response variables are typical for social science applications. For numerical responses usually a linear regression model is used, where the expected value of the response variable is linked to a linear combination of the predictor variables. For binary responses the expected value is a probability, which is linked to the linear predictor by the logistic link function. Finally, for ordinal response variables, researchers typically use the cumulative logistic regression model (Agresti, 2013; McCullagh, 1980). One interpretation of this ordinal regression model is based on an underlying continuous latent variable that is partitioned in a set of ordered classes using a set of thresholds or cutpoints.

The reduced rank model has been generalized for non‐numeric response variables. Yee (2015) extended the models for response variables in the exponential family, whereas De Rooij (2023) developed an extension specifically for binary response variables. For ordinal variables, De Rooij et al. (2025), recently described a reduced rank model with a cumulative link function.

In the multivariate social context defined above, both the response and the predictor variables may have mixed measurement levels. The reduced rank models mentioned in the previous paragraph consider one type of response variables, that is, all responses should be numeric or all should be binary or all should be ordinal. In empirical research, however, typically the responses have different measurement scales. In the example considered in Section 7, for example, there are binary and ordinal response variables. Luo et al. (2018) developed a reduced rank model for mixed types of responses. They did consider numeric, binary and count response variables, but not ordinal ones. In the social sciences, ordinal response variables are most common. Furthermore, their method only allows for numeric predictor variables. Oftentimes, however, categorical predictor variables are collected in the social sciences.

Different measurement levels of predictors are usually taken into account by creating a set of dummy variables. Specifically, for ordinal variables this dummy approach does not take into account the nature of the variable. We will include optimal scaling of the predictor variables. On the predictor side of the model, we will consider variables that might be numeric, ordinal, nominal or binary. To take the measurement level into account, optimal scaling can be employed (Gifi, 1990). In optimal scaling, a categorical variable is replaced by a set of quantifications, that are transformations (i.e. a scaling) of a variable $\phi_{p}=\varphi_{p}(x_{p})$. The transformation functions, $\varphi_{p}(\cdot )$, are variable specific and take into account the measurement level of the variable. The result of the transformation of variable $x_{p}$ is a new, optimally scaled, variable $\phi_{p}$, optimal in the sense that the transformation minimizes a loss function. Regression with optimal scaling was first proposed by Young et al. (1976). A detailed treatment was recently given by Meulman et al. (2019). Willems (2020) proposed to use optimal scaling of categorical predictors in generalized linear models and survival analysis.

In this manuscript, we will develop a reduced rank regression model for mixed numeric, ordinal and binary response variables with optimal scaling for the predictor variables. Therefore, we combine the work presented in De Rooij (2023) and De Rooij et al. (2025) for binary and ordinal response variables and expand it with numeric response variables. Furthermore, we add optimal scaling of predictor variables to the framework, a feature not present in these two papers. In Section 2, we present the model and its interpretation. In Section 3, we present a majorization‐minimization (MM) algorithm for maximum likelihood estimation of the parameters of the model. In Section 4, we investigate using a simulation study the behaviour of the algorithm under different scenarios. In Section 5, we describe model selection issues in detail. In Section 6, we look further at the performance of the model under different notions of true rank and sample size. In Section 7, we will describe an application of the methodology to survey data obtained through the Eurobarometer studies, where the responses are a mixture of binary and ordinal variables, and the predictors a mixture of numerical, nominal and ordinal variables. We end this paper with a general discussion and conclusion.

## 2 GENERALIZED MIXED REDUCED RANK REGRESSION

We will have a set of P predictor and R response variables. The response variables are indexed by r (r=1…,R), while the predictors are indexed by p (p=1…,P). The data are collected for N participants. The observations for participant i (i=1…,N) are denoted by $y_{i}$ and $x_{i}$, for the responses and predictors, respectively.

In what follows, we first describe the treatment of predictor variables after which we describe how we treat the different types of response variables. We end this section with the likelihood equations for our model.

### 2.1 Predictor Variables

We have P predictor variables, that are partitioned in a numeric set (${𝒩}_{p}$) and another set with discrete predictor variables, that are nominal, ordinal and binary predictor variables (${𝒟}_{p}$). A subset of the discrete predictors are the ordinal predictors, indicated by ${𝒪}_{p}$. For the numeric variables, we quantify them by standardizing the values, that is

$$\phi_{p}=\frac{x_{p}-{\bar{x}}_{p}}{\text{sd}(x_{p})},$$

where sd() computes the standard deviation.

For the discrete (non‐numeric) predictor variables, indicator matrices $G_{p}$ of size $N\times C_{p}$, where $C_{p}$ is the number of categories of predictor p, are defined. An optimally scaled variable is obtained by

$$\varphi_{p}(x_{p})=G_{p}w_{p}$$

where $w_{p}$ are quantifications, to be estimated. The quantifications are estimated such that they minimize the loss function (i.e. the negative log likelihood, see next Section). The $w_{p}$ obtained in this way, are optimal for binary and nominal predictor variables. For the subset of ordinal predictor variables (${𝒪}_{p}$) an extra step is needed, as the quantifications may not be ordered correctly. Therefore, the unconstrained quantifications are projected on the cone of admissible transformations. For an ordinal scaling level, this amounts to performing a monotone regression (Busing, 2022; De Leeuw, 2005). After finding the optimal scaling quantifications, a rescaling is done such that the optimal scaled variables are standardized, that is, they are centred and have variance equal to one.

The optimal scaling for nominal and ordinal variables as outlined above is called *single nominal* and *single ordinal* in Gifi (1990). Let us look a bit more in detail what this entails. Suppose, we have a simple regression model with a numeric outcome and a categorical predictor variable with three categories. The standard approach is to create two dummy variables in this case. Let $d_{1}$ be the first dummy and $d_{2}$ be the second, so that the regression model could be written as $y=m+b_{1}d_{1}+b_{2}d_{2}+\epsilon$. Define $\eta =b_{1}d_{1}+b_{2}d_{2}$ and denote by $\bar{\eta }$ and sd(η) the average and standard deviation of η. Finally, denote ϕ the z‐scores of η, such that we may rewrite the regression with dummy model as

$$y=m+b_{1}d_{1}+b_{2}d_{2}+\epsilon =(m+\bar{\eta })+\text{sd}(\eta )\phi +\epsilon .$$

Note that ϕ is a transformation function of the original categorical predictor variable x, that is ϕ=φ(x). Therefore, we may write

$$y=m^{*}+b\varphi (x)+\epsilon$$

which is the optimally scaled equivalent of the regression with dummy variables, where $m^{*}=m+\bar{\eta }$ and b=sd(η). This shows the two models are equivalent for regression with a single outcome. It is this property that we like to generalize in the mixed reduced rank regression models, a model for multiple response variables and multiple predictors. We intent to find one set of quantifications of a categorical predictor variable that is jointly optimal for predicting all responses. Finally note that for binary predictor variables, the optimally scaled variant is simply equal to the z‐scores of the original dummy variable.

The transformed predictor variables will be collected in the N×P matrix Φ,

$$\Phi =\left[\phi_{1},\text{…},\phi_{P}\right]=\left[\varphi_{1}(x_{1}),\text{…},\varphi_{P}(x_{P})\right].$$

The elements of a row of the matrix Φ will be collected in the P‐dimensional column vector $\phi_{i}$, representing the optimally transformed variables for observation i (i=1,…,N).

### 2.2 Response Variables

The R response variables (r=1…,R) are partitioned in three sets: a set of numeric variables 𝒩, a set of binary variables ℬ, and a set of ordinal variables 𝒪.

We define the canonical term $\theta_{ir}$ and the following bilinear or reduced rank structure is imposed

$$\theta_{ir}=m_{r}+\phi_{i}^{\prime }Bv_{r},$$

where $m_{r}$ is an intercept, $\phi_{i}$ are the optimally scaled predictor values for participant i, B are regression weights to be estimated and $v_{r}$ are loadings for the r‐th response variable. The matrix B is of size P×S and the vector of loadings for response variable r has length S. This number has to be chosen by the researcher, it is the required rank or *dimensionality*. The loadings $v_{r}$ can be collected in the R×S matrix V as

$$V={\left[v_{1},\text{…},v_{r},\text{…},v_{R}\right]}^{\prime }.$$

For identification, we require the matrix V to be orthogonal, that is $V^{\prime }V=I$, where I is the identity matrix of order S. Furthermore, we require $U^{\prime }U$ to be a diagonal matrix, where U=ΦB.

For numeric responses, $\theta_{ir}$ represents the expected or estimated value of the response r. We assume a normal distribution for the numeric response variables.

Similarly, for binary response variables, $\theta_{ir}$ represent the log‐odds form, that is,

$$\log\frac{\pi_{ir}}{1-\pi_{ir}}=\theta_{ir}.$$

We assume the binary response variables to have a Bernoulli distribution with probability $\pi_{ir}$.

For ordinal variables, the story is a bit different. The number of categories of response variable r is $C_{r}$, coded as $c=1,\text{…},C_{r}$. Underlying each ordered categorical response variable $y_{r}$ we assume a continuous latent variable $y_{r}^{*}$. These latent variables are modelled as

$$y_{ir}^{*}=\theta_{ir}+\epsilon_{ir},$$

where $\theta_{ir}$, the canonical parameter, is defined as

$$\theta_{ir}=\phi_{i}^{\prime }Bv_{r}.$$

We see that for ordinal response variables the $m_{r}=0$, because, without loss of generality, we can assume that the latent underlying continuous response variable is centred, so no intercept is needed. The continuous underlying variable is partitioned through a set of cut‐points or *thresholds* to form a set of ordered categories. Let $-\infty =t_{0}<t_{1}<\text{…}<t_{C_{r}}=\infty$ define the set of thresholds such that an observed ordinal response $y_{r}$ satisfies

$$y_{r}=c\;\;\text{if}\;t_{c-1}\leq y_{r}^{*}<t_{c},$$

for $c=1,\text{…},C_{r}$. It is typically assumed that the $\epsilon_{ir}$ are independent and identically distributed error terms following a cumulative logistic distribution F(·), that is,

$$F(\eta )=\frac{1}{1+\exp(-\eta )}\;\text{for}\;\eta \in \;(-\infty ,\infty ).$$

It follows that

$$F^{-1}\left(P(y_{ir}\leq c)\right)=\log\left(\frac{P(y_{ir}\leq c)}{P(y_{ir}>c)}\right)=t_{r_{c}}-\theta_{ir},$$

where, similar to the proportional odds regression model (Anderson & Philips, 1981; McCullagh, 1980), the thresholds are *category specific* but the structural part of the model is *variable specific*. From this specification of the model for ordinal variables, it follows that the probability that person i will respond with category c on response variable r is

$$\pi_{irc}=P(y_{ir}\leq c)-P(y_{ir}\leq c-1),\;\text{for}\;r\in 𝒪,$$

a probability that we need to define the log‐likelihood function. The ordered response follows a multinomial distribution with probabilities $\pi_{irc}$.

Summarizing, for numeric, binary and ordinal response variables the canonical parameters are defined in terms of a conditional mean or intercept (m), the regression weights (B) and the loadings (V). The intercepts for ordinal variables are, by definition, equal to zero. Instead, for these ordinal variables thresholds are defined (t) that partition the latent underlying continuous variable into an ordinal categorical observed variable.

### 2.3 Log‐likelihood function

The parameters of the generalized mixed reduced rank models are estimated by maximum likelihood. We will denote the negative of the log‐likelihood function by ℒ(θ,t). The canonical parameters (θ), will be later parameterized by the intercepts (m), weights (B), loadings (V) and the quantifications (w). The vector t collects the set of threshold parameters for ordinal response variables.

We assume conditional independence, that is, given the low rank representation the response variables are independent, such that the negative log likelihood partitions in contributions of the single response variables, that is,

$$ℒ(\theta ,t)=\underset{r}{\sum }{ℒ}_{r}(\theta ,t),$$

where ${ℒ}_{r}(\theta ,t)$ depends on the set of response variable r, that is

$${ℒ}_{r}(\theta ,t)=\left\{\begin{array}{lr} \underset{i}{\sum }\frac{1}{2\sigma^{2}}{\left(y_{ir}-\theta_{ir}\right)}^{2}+N\log(\sqrt{2\pi \sigma^{2}}) & \text{if}\;r\in 𝒩\\ \underset{i}{\sum }-\log({\left(1+\exp(-q_{ir}\theta_{ir})\right)}^{-1}) & \text{if}\;r\in ℬ\\ \underset{i}{\sum }\underset{c}{\sum }-g_{irc}\log\pi_{irc} & \text{if}\;r\in 𝒪 \end{array}\right.,$$

where the expression for the binary variables was derived in De Leeuw (2006) with $q_{ir}=2y_{ir}-1$. In the log‐likelihood for ordinal response variables we use the indicator $g_{irc}$ which equals one when person i on ordinal response variable r responds with category c and zero otherwise.

## 3 ALGORITHM

The general algorithm will alternate between updating the canonical part and the threshold parameters. When updating the canonical parameters, we assume that the threshold parameters are fixed and *vice versa*. To avoid cluttering of notation, we will write ℒ(θ)=ℒ(θ,t) when we update the canonical parameter and ℒ(t)=ℒ(θ,t) when updating the thresholds.

In the canonical part of the algorithm there are orthonormality constraints on the parameters V and orthogonality constraints on U=ΦB. Since orthonormal constraints are not convex (Landgraf & Lee, 2020; Wen & Yin, 2013) finding a global solution for our model is infeasible in most scenarios using standard algorithms (like Newton or coordinate descent algorithms). To deal with the difficulties imposed by orthonormality, we use an MM algorithm. MM stands for Majorization Minimization (Heiser, 1995; Hunter & Lange, 2004; Nguyen, 2017). The concept behind MM, applied to finding a minimum of the function ℒ(θ), with θ representing a vector of parameters, involves defining an auxiliary function known as a *majorization function*, ℳ(θ|ϑ). The vector ϑ is a vector with so‐called support points of the same length as the vector θ. In the iterative algorithm, the support points are usually given by the values of the parameters at that stage in the algorithm. The majorization function has two key characteristic

ℒ(ϑ)=ℳ(ϑ|ϑ)

and

ℒ(θ)≤ℳ(θ|ϑ).

These equations indicate that ℳ(θ|ϑ) is a function positioned above (i.e. majorizes) the original function, touching it at the supporting point. An iterative algorithm can be formulated as

$$ℒ(\theta^{+})\leq ℳ(\theta^{+}|\vartheta )\leq ℳ(\vartheta |\vartheta )=ℒ(\vartheta ),$$

where $\theta^{+}$ is determined as

$$\theta^{+}={\text{argmin}}_{\theta }\;ℳ(\theta |\vartheta )\;,$$

representing the updated parameter, which becomes the ϑ in the next iteration.

MM algorithms have several properties. Usually MM algorithms have a simple numerical minimization method. Whereas Newton type algorithms often require the computation of an inverse of a relatively large matrix in every iteration, MM algorithms might avoid such computationally heavy inverses. We will see shortly that in our case the majorization function is a least‐squares problem that is relatively easy to solve. Furthermore, the value of the loss function (i.e. in our case the negative log‐likelihood) should never increase, which makes it easy to check the programming. MM algorithms are globally convergent and usually end in a local minimum. The disadvantages of MM algorithms are that they are slow (linear convergence rate) and it may be difficult to prove convergence of the parameters.

### 3.1 MM algorithm for canonical part

The negative log likelihood for response variable r is defined as a sum over individual parts, that is

$${ℒ}_{r}(\theta )=\overset{N}{\underset{i=1}{\sum }}{ℒ}_{ir}(\theta_{ir}).$$

Finding a majorization function for each ${ℒ}_{ir}(\theta_{ir})$ also gives a majorization function for the sum. Looking at a single element, where we omit the subscripts for the moment, the *quadratic majorization theorem* states that

$$ℒ(\theta )\leq ℒ(\vartheta )+\frac{\partial ℒ(\theta )}{\partial \theta }(\theta -\vartheta )+\frac{1}{2}(\theta -\vartheta )\kappa (\theta -\vartheta )=ℳ(\theta |\vartheta )$$

for any $\kappa \geq \psi =\frac{\partial^{2}ℒ(\theta )}{\partial \theta^{2}}$ (Hunter & Lange, 2004).

Denote $\xi =\frac{\partial ℒ(\theta )}{\partial \theta }$, and let us rewrite step by step the majorization function ℳ(θ|ϑ), that is

$$\begin{array}{cc} ℳ(\theta ,\vartheta ) & =ℒ(\vartheta )+\xi (\theta -\vartheta )+\frac{1}{2}(\theta -\vartheta )\kappa (\theta -\vartheta )\\ & =ℒ(\vartheta )+\xi \theta -\xi \vartheta +\frac{\kappa }{2}(\theta^{2}+\vartheta^{2}-2\theta \vartheta )\\ & =\frac{\kappa }{2}\theta^{2}+2\frac{\kappa }{2}\theta \left(\frac{1}{\kappa }\xi -\vartheta \right)+c_{1}\\ & =\frac{\kappa }{2}\theta^{2}-2\frac{\kappa }{2}\theta \left(\vartheta -\frac{1}{\kappa }\xi \right)+c_{1}\\ & =\frac{\kappa }{2}\left(\theta^{2}-2z\theta \right)+c_{1}\\ & =\frac{\kappa }{2}\left(\theta^{2}-2z\theta +z^{2}\right)-\frac{\kappa }{2}z^{2}+c_{1}\\ & =\frac{\kappa }{2}{\left(\theta -z\right)}^{2}-\frac{\kappa }{2}z^{2}+c_{1}\\ & =\frac{\kappa }{2}{\left(\theta -z\right)}^{2}+c \end{array}$$

where $z=(\vartheta -\frac{1}{\kappa }\xi )$, a working response and $c=c_{1}-\frac{\kappa }{2}z^{2}$ with $c_{1}=ℒ(\vartheta )-\xi \vartheta +\frac{\kappa }{2}\vartheta^{2}$, all constants with respect to θ. The last line shows that the majorization function is a least‐squares function, it takes the squared difference between the parameter θ and the working response z. For the different types of response variables, we have to derive the expression of the first derivatives ξ and the majorizing constant κ.

#### 3.1.1 Majorization function for numeric response variables

The loss function is

$${ℒ}_{ir}(\theta_{ir})=\frac{1}{2\sigma^{2}}{\left(y_{ir}-\theta_{ir}\right)}^{2}+N\log(\sqrt{2\pi \sigma^{2}}).$$

The first derivative of ${ℒ}_{ir}(\theta_{ir})$ with respect to $\theta_{ir}$ is

$$\begin{array}{c} \xi_{ir}\equiv \frac{\partial {ℒ}_{ir}(\theta_{ir})}{\partial \theta_{ir}}=\frac{1}{\sigma^{2}}(\theta_{ir}-y_{ir}) \end{array}$$

and the second derivative is

$$\psi_{ir}\equiv \frac{\partial^{2}{ℒ}_{ir}(\theta_{ir})}{\partial \theta_{ir}^{2}}=\frac{1}{\sigma^{2}}$$

so that an upper bound is obtained for any $\kappa \geq \sigma^{-2}$. This majorization function was also used by Song et al. (2021) in their approach for principal component analysis of binary and numeric variables.

#### 3.1.2 Majorization function for binary response variables

The loss function is

$${ℒ}_{ir}(\theta_{ir})=-\log\frac{1}{1+\exp(-q_{ir}\theta_{ir})}.$$

The first derivative of ${ℒ}_{ir}(\theta_{ir})$ with respect to $\theta_{ir}$ is

$$\begin{array}{cc} \xi_{ir}\equiv \frac{\partial {ℒ}_{ir}(\theta_{ir})}{\partial \theta_{ir}} & =-(y_{ir}-\pi_{ir}) \end{array}$$

and the second derivative is

$$\psi_{ir}\equiv \frac{\partial^{2}{ℒ}_{ir}(\theta_{ir})}{\partial \theta_{ir}^{2}}=\pi_{ir}(1-\pi_{ir})$$

so that an upper bound is obtained for any $\kappa \geq \frac{1}{4}$. This majorization function was derived by De Leeuw (2006) and was also used in the reduced rank algorithm for binary responses in De Rooij (2023).

#### 3.1.3 Majorization function for ordinal response variables

For ordinal variables, we start out a bit different because we deal with a latent variable and therefore aim for an EM algorithm. In the EM algorithm, the first step is to define the *complete data negative log‐likelihood*, that is, the likelihood assuming that we have observed the underlying latent variable. In the E‐step, the expected value of this complete data negative log‐likelihood is obtained, which in the M‐step is minimized. For minimization, we use an upper bound again like in the MM algorithm.

An element of the complete data negative log‐likelihood is

$${ℒ}_{ir}^{c}(\theta_{ir})=-\log f(y_{ir}^{*}-\theta_{ir}),$$

where f(·) is the probability density function of the logistic distribution. The conditional expectation of the second‐order Taylor expansion of the complete data negative log‐likelihood around the current value ϑ is

$$\begin{array}{c} 𝔼({ℒ}_{r}^{c}(\theta_{ir}))=𝔼({ℒ}_{r}^{c}(\vartheta_{ir}))+(\theta_{ir}-\vartheta_{ir})𝔼\left(\frac{\partial {ℒ}_{r}^{c}(\vartheta_{ir})}{\partial \theta_{ir}}\right)+\frac{1}{2}(\theta_{ir}-\vartheta_{ir})𝔼\left(\frac{\partial^{2}{ℒ}_{r}^{c}(\vartheta_{ir})}{\partial^{2}\theta_{ir}}\right)(\theta_{ir}-\vartheta_{ir}). \end{array}$$

Let us define $p_{ir}=\frac{1}{1+\exp(-y_{ir}^{*}+\theta_{ir})}$ so that $\log f(y_{ir}^{*}-\theta_{ir})=\log p_{ir}(1-p_{ir})$. The partial derivative is

$$\frac{\partial {ℒ}_{r}^{c}(\vartheta_{ir})}{\partial \theta_{ir}}=-\frac{\partial \log f(y_{ir}^{*}-\theta_{ir})}{\partial \theta_{ir}}=1-2p_{ir}.$$

A closed form expression for the expectation of $p_{ir}$ is (De Rooij et al., 2025; Jiao, 2016)

$$\begin{array}{c} \begin{array}{ll} 𝔼(p|y,\theta ,t) & =\left\{\begin{array}{cc} \left[\frac{\exp(2t_{y}-2\theta )}{2{\left[\exp(t_{y}-\theta )+1\right]}^{2}}\right]/F(t_{y}-\theta ) & \text{if}\;y=1\\ \left[\frac{2\exp(t_{\left(y-1\right)}-\theta )+1}{2{\left[\exp(t_{\left(y-1\right)}-\theta )+1\right]}^{2}}-\frac{2\exp(t_{y}-\theta )}{2{\left[\exp(t_{y}-\theta )+1\right]}^{2}}\right]/\left(F(t_{y}-\theta )-F(t_{\left(y-1\right)}-\theta )\right) & \text{if}\;2\leq y<C\\ \left[\frac{2\exp(t_{\left(y-1\right)}-\theta )+1}{2{\left[\exp(t_{\left(y-1\right)}-\theta )+1\right]}^{2}}\right]/\left(1-F(t_{\left(y-1\right)}-\theta )\right) & \text{if}\;y=C \end{array}\right.\; \end{array} \end{array}$$

where we used y and θ instead of $y_{ir}$ and $\theta_{ir}$ for readibility. The expectation has to be evaluated at the current parameter estimates θ and t. Let us denote by $\xi_{ir}$ the expected value of the first derivative, that is

$$\xi_{ir}=1-2𝔼(p_{ir}|y_{ir},\theta_{ir},t_{r}).$$

An upper bound for the (expectation of the) second derivative is given by any κ≥1/4. The ξ and κ can be used in the majorization function. This majorization function was derived by De Rooij et al. (2025).

#### 3.1.4 Combining majorization functions

Our negative log‐likelihood function is

$$ℒ(\theta )=\underset{r}{\sum }{ℒ}_{r}(\theta )=\overset{N}{\underset{i=1}{\sum }}\overset{R}{\underset{r=1}{\sum }}{ℒ}_{ir}(\theta_{ir}).$$

We derived majorization functions for ${ℒ}_{ir}(\theta_{ir})$ for numeric, binary and ordinal response variables, each having a least squares form. Because majorization is closed under summation, we have

ℒ(θ)≤ℳ(θ,ϑ),

where

$$ℳ(\theta |\vartheta )=\sum_{i}\sum_{r}ℳ(\theta_{ir}|\vartheta_{ir})=\|Z-1m^{\prime }-\Phi BV^{\prime }{\|}^{2},$$

is a least squares function. The matrix Z has elements $z_{ir}=\vartheta_{ir}-\frac{1}{\kappa^{*}}\xi_{ir}$, where $\kappa^{*}=\max(\frac{1}{4},\sigma^{-2})$. The vector m contains the $m_{r}$ for r∈{𝒩,𝒟} and zeros for r∈𝒪.

The choice of $\kappa^{*}=\max(\frac{1}{4},\sigma^{-2})$ works fine except when the variance $\sigma^{2}$ is close to zero. As we do not know the value of this variance, we estimate it from the data in each iteration of the algorithm (see Section 3.1.4) and plug the estimate in the algorithm. The estimate will be close to zero when the numeric response variable are approximated very well. In that case, $\kappa^{*}$ becomes a very large value and the working responses stay very close to the expected value of the previous iteration. In that case, the algorithm gets stuck. In our software, we implemented a warning when the estimated variance becomes very small, that is, ${\hat{\sigma }}^{2}<0.05$.

#### 3.1.5 Update of the Regression Weights

To update the regression weights B, we first define the auxiliary matrix $\tilde{Z}=Z-{1m}^{\prime }$. The least squares loss function can be written as

$$\|\tilde{Z}-\Phi {\mathrm{BV}}^{\prime }{\|}^{2}=\|\text{Vec}(\tilde{Z})-\left(V⊗\Phi \right)\text{Vec}(B){\|}^{2}=\|\tilde{z}-Hb{\|}^{2},$$

where $\tilde{z}=\text{Vec}(\tilde{Z})$, b=Vec(B) and H=V⊗Φ. This is a standard regression problem such that

$$b^{+}={\left(H^{\prime }H\right)}^{-1}H\tilde{z}.$$

The computational burden can be reduced by noting that $H^{\prime }H=I_{S}⊗\Phi^{\prime }\Phi$, simplifying the computation of the inverse.

#### 3.1.6 Update of the Loadings

To update the loadings, we use the same auxiliary matrix $\tilde{Z}$ as above and need to minimize

$$\|\tilde{Z}-\Phi {\mathrm{BV}}^{\prime }{\|}^{2},$$

under the restriction $V^{\prime }V=I$. This function can easily be minimized using the lower bounds described in Ten Berge (1993). Therefore, define the singular value decomposition

$$B^{\prime }\Phi^{\prime }\tilde{Z}=P\Delta Q^{\prime },$$

such that an update for V is given by

$$V^{+}=Q_{s}P_{S}^{\prime },$$

where $Q_{S}$ denote the singular vectors corresponding to the S largest singular values and similarly for $P_{S}$.

#### 3.1.7 Update of the intercepts

To update the intercepts for numeric and binary response variables, we define the auxiliary matrix $\tilde{Z}=Z-\Phi {\mathrm{BV}}^{\prime }$, from which we only retain the columns for numeric and binary responses. With this auxiliary matrix, we need to minimize

$$\|\tilde{Z}-{1m}^{\prime }{\|}^{2}.$$

The solution is

$$m^{+}={\tilde{Z}}^{\prime }1{\left(1^{\prime }1\right)}^{-1}.$$

#### 3.1.8 Update of Residual Variance

For updating $\sigma^{2}$, we only need the numeric responses. We therefore only focus on the columns related to r∈𝒩. For these columns, we compute the residuals

$$E=\tilde{Z}-1m^{\prime }-\Phi {\mathrm{BV}}^{\prime },$$

for which we compute the variance to obtain an update of $\sigma^{2}$ as

$$\frac{1}{N\cdot R_{𝒩}-1}\overset{N}{\underset{i=1}{\sum }}\underset{r\in 𝒩}{\sum }e_{ir}^{2},$$

where N is the sample size and $R_{𝒩}$ is the number of numeric response variables.

#### 3.1.9 Update of the Quantifications

For categorical predictor variables, we optimally scale the levels. For numeric variables, the quantified variable is simply the standardized predictor, as discussed in Section 2, which remains constant throughout iterations. The relevant part of the majorization function for the transformations is

$$\|Z-1m^{\prime }-\Phi A{\|}^{2}=\|Z-1m^{\prime }-\phi_{p}a_{p}^{\prime }-\Phi_{\left(-p\right)}A_{\left(-p\right)}{\|}^{2},$$

where $A={\mathrm{BV}}^{\prime }$, $\phi_{p}$ is the p‐th column of Φ, $a_{p}$ is the column vector with the elements of the p‐th row of A, $\Phi_{\left(-p\right)}$ is the matrix Φ without the p‐th column, $A_{\left(-p\right)}$ is the matrix A without the p‐th row.

To find the transformation $\phi_{p}=\varphi_{p}(x_{p})$, we define the indicator matrix $G_{p}$ of size $N\times C_{p}$ and the vector of quantifications $w_{p}$ of length $C_{p}$, so that we can write the optimally quantified variable as

$$\phi_{p}=\varphi_{p}(x_{p})=G_{p}w_{p},$$

With—depending on the scaling level—constraints on $w_{p}$. Let us first define the auxiliary matrix $\tilde{Z}=Z-1m^{\prime }-\Phi_{\left(-p\right)}A_{\left(-p\right)}$ then the majorization function becomes

$$\|\text{Vec}(\tilde{Z})-\left(a_{p}⊗G_{p}\right)w_{p}{\|}^{2},$$

a simple regression problem. Defining, $Q=a_{p}⊗G_{p}$ the unconstrained update is

$$w_{p}^{+}={\left(Q^{\prime }Q\right)}^{-1}Q^{\prime }\tilde{z}.$$

For $p\in {𝒪}_{p}$, the ordinal predictor variables, we need an extra step. In this extra step this update is projected onto the cone of admissible quantifications (Meulman et al., 2019), that is, the quantifications should have the correct order either increasing or decreasing. This amounts to a weighted monotone regression (Busing, 2022; De Leeuw, 2005) of the $w_{p}^{+}$ with weights equal to the observed frequencies of each of the response categories. Because the relationship can either be monotone increasing or monotone decreasing, we perform two of such monotone regressions and select the one that fits best. Note that these monotone regression problems are very small, as they only involve the number of categories of the ordered predictor variable.

In the last step of the optimal scaling process, we rescale $w_{p}$ such that the mean of $\phi_{p}$ is equal to zero and its variance equal to one. This is important, as we multiply the optimally scaled variable with a regression weight and without standardizing we would not be able to obtain unique estimates.

### 3.2 Estimation of thresholds

The thresholds of the ordinal response variables are not part of the canonical parameters. Where above we focused on minimizing ℒ(θ), that is, ℒ(θ,t) with t fixed, now we will focus on minimizing ℒ(t), that is, ℒ(θ,t) with θ fixed. Because of the local independence assumption, we can estimate the thresholds separately for each response variable. For estimation of the thresholds for response variable r∈𝒪, we use standard maximum likelihood estimation where we fixed the other estimates.

### 3.3 Summary

A summary of our algorithm can be found in Algorithm 1. As input we need the response variables and the predictor variables an information about the measurement levels of these variables. The algorithm needs starting values (line 3) that we derive using a standard reduced rank regression model treating all predictors and responses as numeric variables. With the starting values the algorithm goes in a loop where different elements are updated in steps. These steps are repeated until the decrease of the negative log‐likelihood is smaller than a pre‐set convergence criterion $\epsilon_{c}=1$ (e.g. $\epsilon_{c}=1\times 10^{-6}$). The algorithm results in a unique solution. The order of the steps described above can be changed, that would lead to the same estimates.

ALGORITHM 1 **GMR3 algorithm**

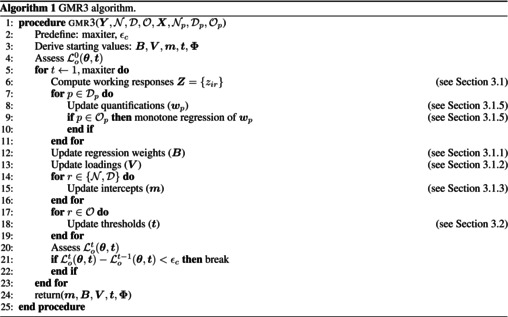

## 4 SIMULATION STUDY

To evaluate our algorithm, we conducted a simulation study. As discussed in the introduction, Luo et al. (2018) proposed a reduced rank model for mixed numeric, binary and count variables response variables and numeric predictors. We propose a reduced rank model for mixed numeric, binary and ordinal response variables and mixed predictor variables. We can therefore compare the two algorithms for a mixture of binary and numeric response variables with numeric predictors.

In our first condition, we followed the set‐up of Luo et al. (2018). We consider their Model 1, a low‐dimensional example, with a few adaptions. We set P=8, R=8 and S=2. Among the responses, four of them are generated from Normal distribution and four from the Bernoulli distribution. The predictor matrix is constructed by generating its entries as independent and identically distributed random samples from the standard normal distribution 𝒩(0,1). The coefficient matrix B is an orthogonal matrix from the QR decomposition of a random matrix filled with 𝒩(0,1) entries, and all entries in V are samples from the uniform distribution 𝒰(−1,1). We set the intercept vector equal to 0. The numeric responses are drawn from the normal distribution with mean equal to the canonical term and variance equal to one. The binary responses are drawn from the Bernoulli distribution.

Our methodology allows for categorical predictors and ordinal responses. To test our algorithm under these circumstances, we added three conditions. First, we changed the measurement level of all eight predictor variables from numeric to ordinal. Changing from numeric to ordinal predictors, we lose information. In this situation optimal scaling of the ordinal predictor variables is employed in our algorithm. We discretized the numeric predictors into ordinal variables with five categories, both in a balanced and unbalanced manner. In the balanced condition, discretization was based on the .2, .4, .6 and .8 quantiles, whereas the .1, .5, .7 and .8 quantiles were used in the unbalanced scenario. For generating the data, we used the average value of the numeric predictors within each of these categories, whereas for data analysis we coded the predictors using the integers 1 to 5.

Second, we investigated different measurement levels of the response variables. In this condition the predictor variables were at the original numeric level. Where in the first condition there were 4 numeric and 4 binary response variables, in this condition we changed to 4 ordinal and 4 binary response variables and to 4 ordinal and 4 numeric response variables. In the condition with ordinal and binary response variables, the amount of information is reduced compared to condition 1, whereas in the condition with ordinal and numeric response variables, the amount of information increases. The ordinal response variables have four categories and were generated by drawing from a multinomial distribution. The thresholds used to derive the probabilities were set to −1, 0 and 1. The numeric and binary response variables were drawn as in condition 1.

Finally, we also added a condition in which we have a mixture of binary, ordinal and numeric predictors and a mixture of binary, ordinal and numeric responses. For both predictors and responses, we have 2 binary ones, 3 ordinal ones and 3 numeric ones. For the ordinal predictors, there are 2 balanced predictors and 1 unbalanced one. For binary predictors, one is balanced and the other unbalanced.

In total, we therefore have seven scenarios divided in three conditions. We make comparisons for three different sample sizes. A small sample size with 250 observations, a medium sample with 500 observations and a large sample size with 1000 observations. To evaluate performance, we compare the population ${\mathrm{BV}}^{\prime }$ with the estimated $\hat{B}{\hat{V}}^{\prime }$ using the Root Mean Squared Error metric (RMSE). The number of replications for each scenario is equal to 250. We depict the RMSE's from the 250 replications using a boxplot.

**FIGURE 1:**
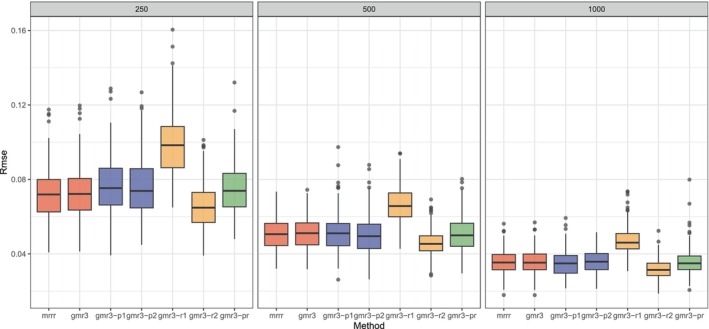
Results of simulation study. Different methods are compared in terms of Root mean squared error (Rmse) under three sample size conditions: small (left, 250), medium (middle, 500) and large (right, 1000). The first method is the mixed reduced rank regression algorithm of Luo and colleagues, the second is our algorithm. These two are applied to data with numeric predictors and a mix of numeric and binary responses. In the third and fourth, we changed the predictors to be ordinal variables, so that the optimal scaling is employed. In the third (gmr3‐p1) the predictor variables are balanced, whereas in the fourth (gmr3‐p2) they are unbalanced. In the fifth and sixth method, we generated ordinal responses: in gmr3‐r1 the responses are a mixture of binary and ordinal responses, whereas in gmr3‐r2 they are a mixture of numeric and ordinal responses. In the last method (gmr3‐pr) both the predictors and responses are a mixture of binary, ordinal and numeric variables.

The results are shown in Figure 1. The figure has three boxes: from left to right for small, medium and large sample sizes. We see, comparing the first two boxplots, that our algorithm performs the same as the algorithm of Luo et al. (2018). The third and fourth boxplots, in purple, show the results when all predictor variables are ordinal. In this case, there is a loss of information and optimal scaling is used for the ordinal predictor variables. We see that this hardly affects the performance of the algorithm. In the fifth and sixth boxplots (in orange) we show the results for the simulations when the measurement level of the responses variables is changed. In the fifth, there is a loss of information compared to the first condition (i.e. first two boxplots) as now we only have binary and ordinal response variables. In the sixth, there is a gain of information compared to the first condition as now we have numeric and ordinal response variables. The final boxplot (in green) shows the results for the case in which both predictors and responses are formed by a mixture of binary, ordinal and numeric responses. The results indicate that the recovery is good in this case.

Comparing the three frames of the figure, we see that with increasing sample sizes, estimation is better, that is, the root mean squared error is lower and the boxes are smaller. The measurement level of the predictor variables does not influence the recovery. The measurement level of the response variables seems to have a larger influence. Specifically in the scenario where there are only binary and ordinal response variables, the root mean squared error is larger than in the other conditions.

As known, the mean squared error (MSE) can be partitioned into squared bias and variance. As can be seen in our graphs, the RMSE in large sample sizes is less than .04, meaning that the MSE is around .0016. If .0016 is the sum of squared bias and variance, then both should be very small. Furthermore, with increasing the sample size, the estimates become better and better, approaching the true values as the RMSE becomes smaller and the boxplots indicate less variability. This suggests that the estimator is consistent.

## 5 MODEL SELECTION FOR GMR^3^

For application of our approach to empirical data model selection is important. In this section, we like to set out our thoughts on model selection for our generalized mixed reduced rank regression model. We discuss several existing methods and how they can be used in our framework.

### 5.1 The overall number of parameters

In the proposed class of models it can be not immediately clear how to determine the overall number of parameters to estimate. This number depends on the number R of response variables, the number P of predictor variables, the required rank S, the number of categories of discrete predictor variables and the number of categories of ordinal response variables.

As described in Section 2, the R response variables are partitioned into the set 𝒩 of numeric variables, the set ℬ of binary variables and the set 𝒪 of ordinal variables. The predictor variables for p=1…,P are divided into two sets: a set of numeric variables ${𝒩}_{p}$ and a set of discrete variables ${𝒟}_{p}$.

For discrete predictors, the number of parameters estimated in optimal scaling is equal to the number of categories (De Leeuw, 2005). However, we restrict the quantifications to having mean zero and variance one, and therefore the number of parameters involved is $C_{p}-2$. For ordinal responses, we estimate the $C_{r}-1$ thresholds, while for numeric and binary responses, we estimate one intercept. The total number of parameters to estimate can be obtained in the following way:

(1) $$𝒦=(P+R-S)\times S+\left[\underset{p\in {𝒟}_{P}}{\sum }C_{p}-2\right]+\underset{r\in \{𝒩,𝒟\}}{\sum }1+\left[\underset{r\in 𝒪}{\sum }C_{r}-1\right]$$

where $C_{p}$ is the number of categories of the p‐th discrete predictor and $C_{r}$ is the number of categories of the r‐th ordinal response variable.

### 5.2 Model selection procedure

The selection of the optimal model requires the selection of both the influential predictors and the optimal dimensionality.^1^

We adopt a two‐step model selection procedure:

I We fix the set of predictors and then select the optimal dimensionality.
II Once the dimensionality is fixed, we verify which predictors are significant.

In general, for maximum likelihood methods there are several type of statistics that can be used for inference. The best known statistics are Wald tests and likelihood ratio tests. For our reduced rank models we need to make the following observations. For Wald statistics, we need standard errors of the parameters. Such standard errors are not a by‐product of our MM‐algorithm. The likelihood ratio statistic compares two nested models. If the model under the null hypothesis is true and certain regularity conditions are satisfied, the likelihood ratio statistic is known to be asymptotically distributed as a chi‐square variable with degrees of freedom equal to the difference in the number of parameters under the two hypotheses. For our models there are two complications: (1) The regularity conditions are not satisfied for selecting the optimal dimensionality, see Takane et al. (2003) and Takane and Van der Heijden (2023) for a detailed discussion; (2) we generally do not belief a certain model to be true as this involves many assumptions.

In contrast, our model can best be cast in the bias‐variance trade‐off where we look for a model that has low bias and low variance. Bias refers to the discrepancy between the true and fitted model, whereas variance refers to the amount by which the fitted model would change if estimated using a different training sample. Complex models have small bias but large variance and usually lead to *overfitting*, the results cannot be generalized to fresh data. Simple models have large bias and small variance, and also cannot be generalized to fresh data because the model is *inadequate*. The goal is to find a model that simultaneously achieves low variance and low bias and therefore minimizes the expected prediction error (Hastie et al., 2009; Vapnik, 1994, 1998, 1999).

For Step I, we identify a set of competing reduced‐rank models with an increasing level of complexity. Section 5.3 describes how to select the complexity of the model such to minimize the expected prediction error when using the model for fresh data with unknown response measurements.

For step II, once the dimensionality is fixed in step I, we compute the (1−α)% bootstrap confidence region of the parameter estimates: the important predictors are those for which the region does not include the origin (i.e. vector with zeros).

### 5.3 The model complexity

Consider K competing models of the set $ℳ=\{{ℳ}_{0},{ℳ}_{1},\text{…},{ℳ}_{k},\text{…},{ℳ}_{K}\}$, all generated by restricting the vector space of parameters in decreasing order to the vector $\theta_{k}$. The number of parameters for model ${ℳ}_{k}$ is given by ${𝒦}_{k}$. The model ${ℳ}_{0}$ is the null model that includes only the intercept and threshold parameters, so there is no contribution of predictors. The number ${𝒦}_{k}$ can also be understood as the *complexity parameter* that governs the trade‐off between bias and variance.

The aim is to find an optimal model ${ℳ}_{k^{*}}$ that yields the smallest prediction error of fresh data with unknown response measurements. The prediction error can be estimated *indirectly* by making an adjustment to the training error to account for bias due to overfitting. Another approach is to *directly* estimate the test error using cross‐validation (Hastie et al., 2009).

### 5.4 Goodness of fit‐based model selection criteria

Akaike Information Criterion (AIC) and the Bayesian Information Criterion (BIC) are commonly used for model selection (Hastie et al., 2009). Another choice is given by the Mc Fadden R‐squared goodness of fit measure (Mc Fadden, 1974). The general idea of these methods is to penalize the goodness‐of‐fit measure of the model, fitted using a training sample, by the number of estimated parameters. It is a matter of evaluating if it is worth to add more parameters with respect to the improvement of goodness of fit a more complex model provides.

McFadden's adjusted R‐squared with respect to the standard R‐squared measure takes into account the complexity of the model in the following way:

(2) $$R_{a}^{2}({ℳ}_{k})=1-\frac{\hat{ℒ}(\theta_{k},t)+{𝒦}_{k}}{\hat{ℒ}(\theta_{0},t)},$$

where $\hat{ℒ}(\theta_{k},t)$ is the minimum achieved of the negative log likelihood for the model ${ℳ}_{k}$ and similarly $\hat{ℒ}(\theta_{0},t)$ is that quantity for the null model ${ℳ}_{0}$. Adjusted McFadden $R_{a}^{2}$ penalizes the goodness of fit of the current model by the number of parameters to be added with respect to the null model.^2^ The optimal choice of the model maximizes $R_{a}^{2}({ℳ}_{k})$ over all k in the set ℳ.

AIC is based on the entropic or information‐theoretic interpretation of the maximum likelihood method as well as the minimization of the Kullback–Leibler (K–L) information quantity. AIC for any model ${ℳ}_{k}$ can be defined as:

(3) $$\mathrm{AIC}({ℳ}_{k})=2\hat{ℒ}(\theta_{k},t)+2{𝒦}_{k}.$$

The first term $2\hat{ℒ}(\theta_{k},t)$ in AIC is twice the negative log likelihood and acts as a measure of lack of fit to the data; consequently, smaller values will be preferred. The second term $2{𝒦}_{k}$ acts as a penalty term which penalizes models having many parameters. The aim is to reach a balance between the lack of fit and the model complexity: models with smaller AIC values indicate a better balance. The optimal model choice minimizes the $\mathrm{AIC}({ℳ}_{k})$ over all k in the set ℳ.

BIC is an alternative to AIC and is based on an asymptotic Bayesian argument.^3^ Having a finite number of models in the set ℳ, selecting the one with the highest marginal log‐likelihood in large samples is equivalent to minimizing the following measure for all k in the set ℳ:

(4) $$\mathrm{BIC}({ℳ}_{k})=2\hat{ℒ}(\theta_{k},t)+\log(N){𝒦}_{k},$$

where N refers to the sample size. The model that minimizes BIC corresponds to the model with the highest posterior probability. Due to the larger penalty of log(N) on the complexity of the model as opposed to 2 for AIC, BIC often selects a sparser model compared to AIC.

### 5.5 Model selection by cross‐validation

Model selection can be performed by a direct estimate of the prediction error through L times repeated V‐Fold Cross‐validation. The sample is repeatedly partitioned into V folds, each in turn being used to evaluate the model fitted using the remaining (V−1) folds. We derive the V‐Fold Cross‐validation estimate of the prediction error for each model ${ℳ}_{k}$ in the set ℳ in the following way:

(5) $$CV({ℳ}_{k})=\frac{1}{L}\overset{L}{\underset{l=1}{\sum }}\frac{1}{V}\overset{V}{\underset{v=1}{\sum }}\frac{1}{n_{v}}\overset{n_{v}}{\underset{i=1}{\sum }}\overset{R}{\underset{r=1}{\sum }}{ℒ}_{v}({{\hat{\theta }}_{k},\hat{t})}_{ir},$$

where ${ℒ}_{v}({{\hat{\theta }}_{k})}_{ir}$ is the loss function for the i‐th individual and the r‐th response variable evaluated in the v‐fold for the model ${ℳ}_{k}$ the parameters of which are estimated using the other (v−1) folds. Typical choices are V=5 or V=10. The optimal model choice is the model that minimizes the $CV({ℳ}_{k})$ over all k in the set ℳ or the one that fits within one‐standard error of the minimum prediction error estimate.

### 5.6 Bootstrap

We use bootstrap (Davison & Hinkley, 1997; Efron, 1979; Efron & Tibshirani, 1986) to obtain confidence regions for the regression weights and loadings. Buja and colleagues (Buja et al., 2019a, 2019b) recently showed that the bootstrap is preferable to standard statistical tests in many empirical studies, specifically when it is not possible to assume that the model specification is true, as required in standard likelihood‐based statistics. For regression models, researchers can choose between randomly drawing pairs, that is, both the explanatory and response variables, or drawing residuals. The latter assumes that the functional form of the regression model is correct, that the errors are identically distributed and that the predictors are fixed (Davison & Hinkley, 1997). For our generalized mixed reduced rank regression method, we draw pairs of sets of explanatory and response variables, to avoid the dependency upon these assumptions. This sampling scheme also takes into account that there are dependencies among the response variables of a participant and the bootstrap automatically adapts to these dependencies. This resembles the so‐called clustered bootstrap for nested or hierarchical data (Deen & de Rooij, 2020; Sherman & le Cessie, 1997).

The balanced bootstrap can be used to ensure that every participant appears exactly ‘the number of bootstrap’ times in the bootstrap samples, in contrast to randomly drawing bootstrap samples from the parent sample. Davison & Hinkley (1997) show that the balanced bootstrap results in an efficiency gain.

Bootstrap confidence ellipses can be visualized by data ellipses, as discussed by Friendly et al. (2013). To verify whether a predictor variable has a significant contribution, we verify whether the ellipse of predictor p includes the origin. Similarly, to verify whether a response is predicted well from the set of predictors, we verify whether the ellipse of the loadings for response r includes the origin of the space.

## 6 SIMULATION STUDY 2

Before, we turn to an empirical application, we like to further investigate the model and algorithm assuming data generated with different true ranks. As the basis for this simulation study, we use the last condition of the first simulation study (see Section 4), that is, the condition in which we have a mixture of binary, ordinal and numeric predictors and a mixture of binary, ordinal and numeric responses. For both predictors and responses, we have 2 binary ones, 3 ordinal ones and 3 numeric ones. For the ordinal predictors, there are 2 balanced predictors and 1 unbalanced one. For binary predictors, one is balanced and the other unbalanced.

In this second simulation study, we generate data under five conditions. In the first condition, the data are generated based on a true rank 2 model (like in Section 4). In the second and third condition the true rank is four, while in the fourth and fifth condition the true rank is seven. For rank 4 and 7, we define a strong and weak condition. In the strong condition, the coefficient matrix B and the matrix V are generated in the same way as in simulation study 1. That is, the coefficient matrix B is an orthogonal matrix from the QR decomposition of a random matrix filled with 𝒩(0,1) entries, and all entries in V are samples from the uniform distribution 𝒰(−1,1). We set the intercept vector equal to 0.

For the weak condition, we alter B and V such that the structure in dimensions 3, 4,… till the true rank is weak. Therefore, we compute the singular value decomposition of ${\mathrm{BV}}^{\prime }$, that is

$$\mathrm{BV}=P\Lambda Q^{\prime }.$$

To obtain the weak structure, we replace all $\lambda_{s}$ for $s=3,\text{…},S_{true}$ with .20. In such a way, the true rank remains the same, however, only the structure in the first two dimensions is strong and the other dimensions add little signal.

We generate data with three sample sizes, 100, 250 and 500. We replicate the procedure 250 times.

We analyse each generated data set with the rank 2, rank 4 and rank 7 model. Our outcome measure is again the RMSE. We expect that under a strong structure, the RMSE is smallest when the fitted rank is equal to the true rank. With lower fitted rank than true rank, the model will be biased, while with higher fitted rank than true rank, the model will have more variability (Anderson, 2002). Both cases lead to an increase in RMSE.

When the structure is weak, however, we expect that the bias will be negligible because the discrepancy is small due to the weakness of the signal in dimensions 3 and higher. Therefore, the model with lower rank than the true rank will perform better, that is, it will have a smaller RMSE than/then the model with true rank. This will be specifically true when the sample size is small.

**FIGURE 2:**
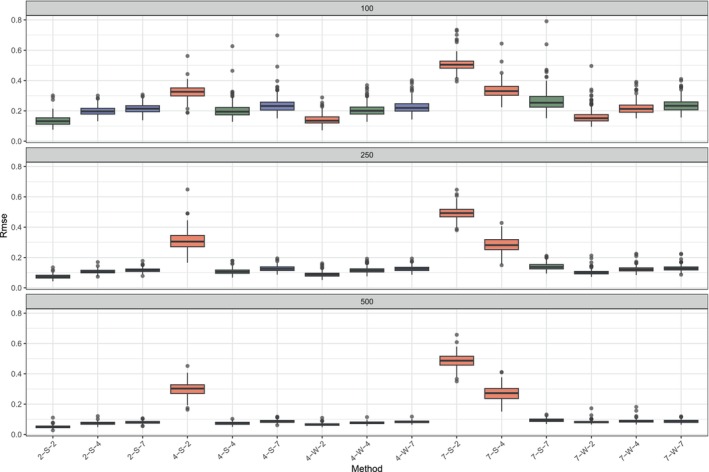
Results of second simulation study. Different methods are compared in terms of Root mean squared error (Rmse) under three sample size conditions: small (upper, 100), medium (middle, 250) and large (lower, 500). The labels on the horizontal axis indicate the data generating condition and the rank of the fitted model. Specifically, 4‐S‐7 denotes the true rank is 4, the structure is strong and a model with rank 7 is fitted; 7‐W‐2 denotes the true rank is 7, but the structure is weak and a rank 2 model is fitted. When the fitted model has a rank lower than the true rank the boxplot is coloured in red, when the two ranks are equal the boxplot is green, whereas when the fitted model has a higher rank than the true rank the colour of the boxplot is blue.

The results are shown in Figure 2 reporting on the horizontal axis the overall 15 conditions and on the vertical axis the RMSE. Specifically, there are five blocks of simulation conditions by fixing the true rank (2, 4, 7, as the first number of the label) and, only for the true rank 4 and 7, the structure Strong or Weak (S, W, as the second character of the label) and varying the rank of the fitted model (2, 4, 7, as the last number of the label). It can be verified from the figure that when the true rank is strong, for each sample size, the fitted model with the corresponding rank has the lowest RMSE. Fitted models with lower rank have higher RMSE because the bias has increased, while fitted models with higher rank have larger RMSE because the variance has increased.

When the generating structure is weak, however, we see a different picture. The results are shown in boxplots 7, 8, 9 and 13, 14, 15. In this case, fitted models with rank lower than the true rank have the smallest RMSE. The results are most clear in the small sample size condition.

A corresponding question is whether the model selection procedure is able to pick the model with the smallest RMSE. Therefore, we look at two of our proposed statistics for dimension/rank selection, the AIC and BIC statistics. For each of the 250 replications, we check which model has the lowest AIC/BIC. The results are shown in Table 1. We see that both the AIC and BIC tend to select the correct rank when the signal is strong. When the signal is weak, both AIC and BIC tend to select the rank 2 model, the models with lowest RMSE. In addition to AIC and BIC, we also use cross validation for dimension selection. It is well known that cross validation minimizes the expected prediction error that breaks down in squared bias and variance (Hastie et al., 2009), that is, (R)MSE.

**TABLE 1:** Number of times the lowest AIC/BIC occurs in 250 replications of the procedure for each of the five blocks of simulation conditions: in bold the number of replications where the true rank is recovered and in italic the modal case.

Simulation	Sample 100		Sample 250		Sample 500	
Condition	AIC	BIC	AIC	BIC	AIC	BIC
2‐S‐2	**244**	**250**	**247**	**250**	**246**	**250**
2‐S‐4	6	0	3	0	4	0
2‐S‐7	0	0	0	0	0	0
4‐S‐2	0	25	0	3	0	0
4‐S‐4	**246**	**225**	**244**	**247**	**248**	**250**
4‐S‐7	4	0	6	0	2	0
4‐W‐2	*234*	*250*	*224*	*250*	*189*	*250*
4‐W‐4	16	0	26	0	60	0
4‐W‐7	0	0	0	0	1	0
7‐S‐2	0	0	0	0	0	0
7‐S‐4	1	67	0	8	0	0
7‐S‐7	**249**	**183**	**250**	**242**	**250**	**250**
7‐W‐2	*217*	*250*	*194*	*250*	80	*250*
7‐W‐4	33	0	55	0	*161*	0
7‐W‐7	0	0	1	0	9	0

## 7 APPLICATION

### 7.1 Eurobarometer Surveys

We will use data from the European Commission's Eurobarometer Surveys of January‐February 2023 (European Commission Brussels, 2023), with a specific focus on residents of the Netherlands, to illustrate our methods. For N=837 Dutch inhabitants we have their opinion on various issues related to Europe, including unification, institutions and policies. We will consider response variables of ordinal and binary type as well as predictors of binary, ordinal and numerical type. For discrete predictor variables, we use optimal scaling to quantify the categories. Hereby the list of variables with their scale of measurement and original coding:

1. Ordinal response variables:
  CI: The interests of the Netherlands are taken into account in the European Union. Ordinal scaled categories: ‘Strongly Disagree’ (SD = 1), ‘Disagree’ (D = 2), ‘Agree’ (A = 3), ‘Strongly Agree’ (SA = 4).
  MW: Every EU member state should have a minimum wage for workers. Ordinal scaled categories: ‘Strongly Disagree’ (SD = 1), ‘Disagree’ (D = 2), ‘Agree’ (A = 3), ‘Strongly Agree’ (SA = 4).
  FS: The EU has taken a series of measures in response to Russia's invasion of Ukraine. To what extent do you agree or disagree with providing financial support to Ukraine? Ordinal scaled categories: ‘Strongly Disagree’ (SD = 1), ‘Disagree’ (D = 2), ‘Agree’ (A = 3), ‘Strongly Agree’ (SA = 4).
  DI: More money needs to be spent on defense in the EU. Ordinal scaled categories: ‘Strongly Disagree’ (SD = 1), ‘Disagree’ (D = 2), ‘Agree’ (A = 3), ‘Strongly Agree’ (SA = 4).
  RE: Reducing oil and gas imports and investing in renewable energy is important for our overall security. Ordinal scaled categories: ‘Strongly Disagree’ (SD = 1), ‘Disagree’ (D = 2), ‘Agree’ (A = 3), ‘Strongly Agree’ (SA = 4).
2. Binary response variables:
  T: Do you rather or do you not have confidence in the European Union? Rather trust = 1, Rather not trust = 0.
  FE: What is your opinion about further expansion of the EU to include other countries in the future? Pro = 1, Against = 0.

1. Binary predictor
  G: Gender (Male = 0, Female = 1).
2. Ordinal predictors:
  PA: Political Alignment with three ordinal categories ‘Left’ = 1, ‘Centre’ = 2, ‘Right’ = 3.
  U: Urbanization with three ordinal categories ‘Rural Area/Village’ = 1, ‘Small/Middle Town’ = 2, ‘Large Town’ = 3).
  E: The highest level of education attained with ordinal categories ‘Pre‐primary’ = 1, ‘Primary’ = 2, ‘Low Secondary School’ = 3, ‘Up Secondary School’ = 4, ‘Post Secondary School’ = 5, ‘Tertiary’ = 6, ‘Bachelor’ = 7, ‘Master’ = 8 and ‘Doctorate’ = 9.
3. Numeric predictor
  A: Age (from 15 to 75 years), standardized to have mean 0 and variance 1.

### 7.2 Model selection

#### 7.2.1 Goodness of fit

Model selection criteria can be based on goodness of fit measures. Both Akaike and Bayesian Information criteria AIC and BIC include the penalization factor accounted for the model complexity as measured by the number of parameters to estimate. For this purpose, Mc Fadden R‐squared measure $R^{2}$ has also the adjusted version $R_{a}^{2}$. All these measures are evaluated for all models with an increasing dimensionality S. With larger dimensionality, the number 𝒦 of parameters to estimate also increases. Model selection results are shown in Table 2. In our application, the BIC suggests a two‐dimensional (i.e. S=2) model with 𝒦=46 parameters whereas, as often is the case, the AIC proposes a less parsimonious three dimensional model with 𝒦=53 parameters. The adjusted Mc Fadden R‐squared measure also suggests three dimensional model, although the differences between the 2, 3, 4 and 5 dimensional models are small.

**TABLE 2:** Model fit statistics for the Eurobarometer data for the set of models including all predictors for different dimensionalities (S).

S	ℒ	𝒦	AIC	BIC	$R_{a}^{2}$
1	5055.49	37	10184.98	10359.98	.033
2	5006.52	46	10105.03	10322.60	.041
3	4990.00	53	10086.01	10336.69	.043
4	4988.96	58	10093.91	10368.24	.042
5	4988.25	61	10098.51	10387.03	.042

#### 7.2.2 Cross validation

**FIGURE 3:**
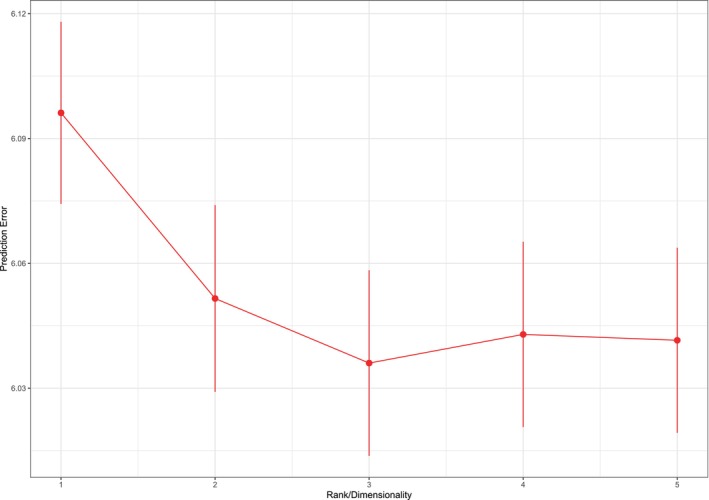
Cross validated prediction error for dimensionalities from one till five. Average prediction error (points) plus and minus one standard error (bars) are shown.

We did ten times repeated 10‐fold cross validation to select the optimal dimensionality (or rank). The results are visualized in Figure 3. The plot shows the average prediction error per participant in the validation sets, against the rank of the model. Also included are error bars that represent the standard error. It can be verified that the rank three model has the lowest prediction error. However, the rank two model (i.e. a more parsimonious model) falls within the one‐standard error range.

From the goodness of fit analysis and the cross validation results, we conclude that the two‐dimensional model provides a good representation of the data.

#### 7.2.3 Bootstrap

**FIGURE 4:**
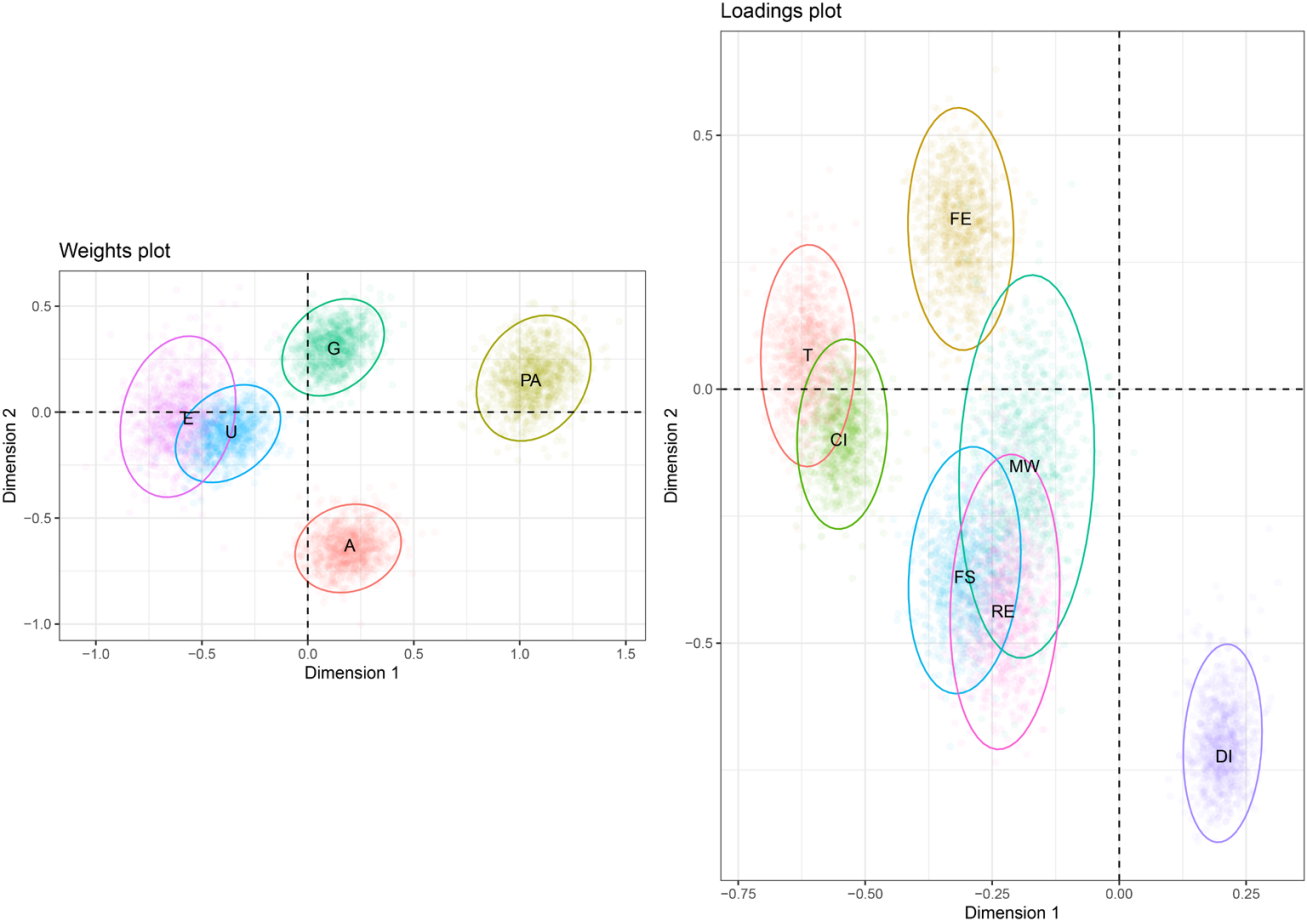
Results of 1000 bootstraps. On the left, the results for the regression weights (B), and on the right the results of the loadings (V). For abbreviations of the variables see Section 7.1.

To investigate the contributions of the predictor variables, we performed a bootstrap analysis. One thousand bootstrap samples were drawn and for each sample the model was fitted. In Figure 4, we show the results of the bootstrap. We separate the information in two graphs, one for the weights (B) and one for the loadings (V).

For the regression weights we can conclude that all predictor variables have a significant contribution in the model, that is, none of the 95% confidence ellipses include the origin. The same conclusion can be drawn for the loadings.

### 7.3 Interpretation

**FIGURE 5:**
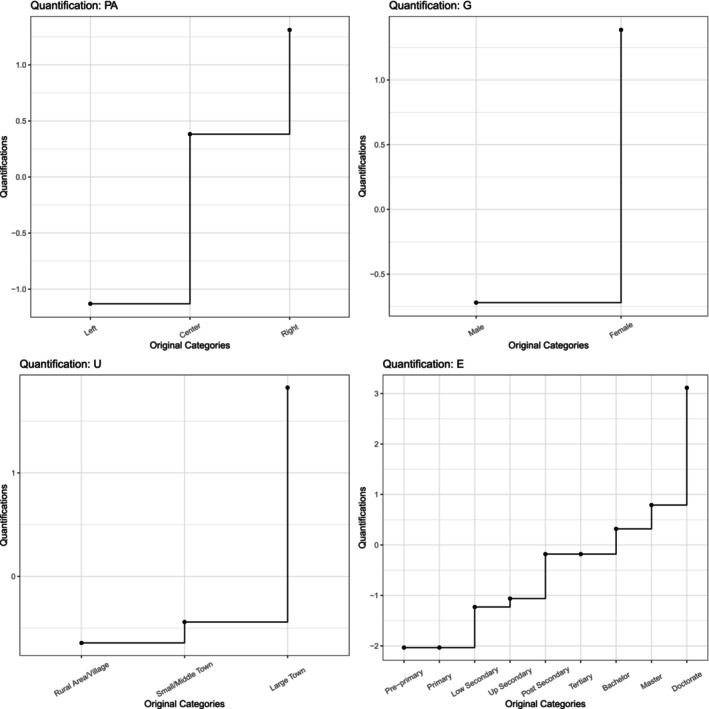
Data transformations of predictors. Nominal or ordinal quantifications on the vertical axis versus original categorical values on the horizontal axis.

We start showing the optimally quantified variables in Figure 5. In this figure, it can be seen that for the gender the quantification for man is −.72, while that for woman is 1.39. For political alignment, the quantifications are −1.13 for left, .36 for middle and 1.31 for right. The difference between left and middle is approximately 1.5 and the difference between right and middle is approximately one unit. These differences can be important for detailed interpretation, as the coefficients are often interpreted as changes in the expected value or the estimated log‐odds with a unit change in the optimally scaled predictor. For urbanization, we see that there is not much difference for the rural area/village (−.64) and the small/middle town (−.44), but that the large town stands out (1.82). For education, we see that the first two levels have about equal quantifications, and also the 3rd and 4th levels have similar quantifications. Then there is a cluster of post secondary, tertiary, bachelor and master, and finally there is a doctoral degree that stands out.

Figure 4, also shows the parameter estimates of the weights and loadings. For example, the regression weights for age (A) are .65 and −.13, which can be verified in the plot. Similarly, the loadings for trust (T) are −.36 and −.50.

We can conclude from Figure 5 that the effects of the predictors urbanization (U) and education (E) are similar, although the effect for education is stronger (larger distance from origin). However, the effect of political allignment (PA) is opposite to that of urbanization (U) and education (E).

For the response variable side of the model, we can see that the predictors influence the two response variables trust (T) and countries interest (CI) in the same way, because their loadings are very similar. The same reasoning holds for the three response variables, minimum wage (MW), financial support for Ukraine (FS) and renewable energy (E). The response variables defence investments (DI) and future enlargement (FE) are a bit isolated, so predictors influence these responses in a different way. The effects on the latter two response variables are in some sense opposite. We see that the reduced rank model implies the responses to be associated.

We can verify that all response variables are described by the predictors to some extent, that is, none of the confidence intervals of the responses includes the origin. Some response variables are closer to the origin (i.e. minimum wage (MW)), whereas others (i.e. countries interest (CI) and trust (T)) are further away from the origin. The distance to the origin represents the strength of discrimination for that response variable.

From the estimates of the regression weights and the loadings, we can compute the implied parameter estimates, $\hat{B}{\hat{V}}^{\prime }$, that is:

**Table:** 

	T	FE	CI	MW	FS	DI	RE
A	−.16	−.27	−.05	.06	.17	.50	.23
PA	−.63	−.28	−.60	−.22	−.38	.11	‐.31
Gr	−.06	.06	−.10	−.07	−.15	−.19	−.16
U	.21	.08	.21	.08	.14	−.01	.12
E	.34	.17	.31	.11	.18	−.10	.14

that shows in the rows the predictor variables and in the columns the response variables. The coefficients can be interpreted as in standard binary or ordinal logistic regression models.

Suppose that we have a 70 year old woman (quantification for age is 1.25 and for woman is 1.39), who is politically left oriented (quantification is −1.13), who lives in a rural area (quantification is −.64) and has a bachelor degree (quantification is .32). From these optimally quantified categories of the predictor variables and the estimated coefficients, we can derive the expected value for the first response variable. Note this first response variable is binary, so we compute

$$\begin{array}{ll} \theta_{i1} & =0.45+1.25\times (-0.16)-1.13\times (-0.63)\;\\ & \;+1.39\times (-0.06)-0.64\times 0.21+0.32\times 0.34=0.85,\; \end{array}$$

where .45 is the estimated intercept for the first variable (not shown before). With this value of the canonical parameter, we can compute the probability that this participant trusts the European Union as an institution, that is,

$$\pi_{i1}=\frac{\exp(0.85)}{1+\exp(0.85)}=0.70$$

Similarly, for variable 3, which is an ordinal variable, we compute

$$\begin{array}{ll} \theta_{i3} & =1.25\times (-0.05)-1.13\times (-0.60)\;\\ & \;+1.39\times (-0.10)-0.64\times 0.21+0.32\times 0.31=0.45\; \end{array}$$

We have to compare this canonical term to the estimated thresholds for this response variable, that is $t_{1}=-2.57$, $t_{2}=-0.91$ and $t_{3}=1.80$. As the canonical parameter falls within the threshold $t_{2}$ and $t_{3}$, we classify this person in class 3, that is, she tends to agree with this question. Alternatively, we can compute the estimated probabilities that this person will respond to each of the four answer categories. These probabilities are .05 for strongly disagree, .16 for disagree, .59 for agree and .21 for strongly agree. Again, this person tends to agree.

Some general conclusions about this Dutch participants that can be derived from the implied coefficients. As the participants grow older (Age), they have less trust in the European Union as an institution, they do not think the EU should be enlarged, believe that their country's interests are not sufficiently respected, tend to agree with a minimum EU wage, the financial support of Ukraine, more defence investments and investments in renewable energy.

As Dutch people are more right wing (PA), they have less trust in the European Union as an institution, they do not think the EU should be enlarged, believe that their country's interests are not sufficiently respected, tend to disagree with a minimum EU wage, the financial support of Ukraine, investments in renewable energy, but tend to agree with more defence investments.

Higher educated participants have more trust in the European Union as an institution, they do think the EU should be enlarged, believe that their country's interests are sufficiently respected, tend to agree with a minimum EU wage, the financial support of Ukraine, investments in renewable energy, but tend to disagree with more defence investments.

### 7.4 Comparison to standard regression models for each of the responses

We also analysed the data by running standard regression methods for each of the responses separately. For Trust in the European Union (T) and further expansion of it (FE), this amounts to fitting binary logistic models and for the others (CI. MW, FS, DI and RE) proportional odds regression models. For the categorical predictor variables, we used dummy coding as is usual, with the first category as the baseline. We estimate the models and use a bootstrap procedure to find the standard errors of the parameters. To make a fair comparison, or our GMR^3^ model, we derived coefficients similar in meaning to those of the separate models. The results are described in detail in Appendix A. Here, we describe some general conclusions.

Although the total negative log‐likelihood is lower than the one obtained with our methodology, the sum of AIC and BIC statistics is higher than the AIC and BIC we obtained. This is mainly because of the number of parameters, which for the separate models equals 115, while for our model it is 46. The estimated coefficients of the separate models do not take into account the ordered nature of the predictors (see Table A1 and compare to Table A2). Some of the estimated coefficients become really large in the separate models, for example, the effects of the dummy variables for eduction on the response variables MW, FS, DI and RE. Such large estimates usually point to very unstable results. The implied corresponding estimates of our model are much better. We can also witness the instability of the estimates in the bootstrap standard errors, shown in Table A3. Some of these standard errors are really huge. As a comparison, we showed the standard errors of the corresponding estimates from our model in Table A4. By fitting one low rank model instead of a separate regression model, we obtain better estimates that are much more stable. Furthermore, from our results in Figure 4 we directly see that the effects from the predictors on, for example, FS and RE are very similar, where such a conclusion is very difficult to obtain from the separate fits.

## 8 CONCLUSIONS AND DISCUSSION

In this paper, we developed a reduced rank regression model for the mixed type of response variables and the mixed type of predictor variables. We named our method GMR^3^, the generalized mixed reduced rank regression model. Earlier work considered reduced rank regression models for a single type of response variables. For numeric responses, the reduced rank model was first described by Anderson (1951) and further developed in the 70s and 80s of the previous century (Anderson, 1951; Davies & Tso, 1982; Izenman, 1975; Tso, 1981; Van den Wollenberg, 1977). Compared to a multivariate regression model, the reduced rank structure is imposed to keep the number of parameters relatively small. The reduced rank model was generalized to response variables of the exponential family (Yee, 2015; Yee & Hastie, 2003), to binary variables (De Rooij, 2023) and to ordinal variables (De Rooij et al., 2025). Hardly any reduced rank model paper considers mixed type of response variables. In this paper, we combined the original numeric model with that for binary and ordinal response variables, to obtain a reduced rank model for a mixture of response variables. These types of variables often occur in the social sciences, like economics, psychology, education, political science, but also health‐related sciences.

Furthermore, we also considered different types of predictor variables, whereas the earlier models could only deal with numeric variables. Therefore, discrete predictor variables have to be coded as dummy variables. We, however, used optimal scaling to quantify the discrete predictors. Whereas there is a lot of knowledge on optimal scaling within least squares problems (see, for example, Gifi, 1990), optimal scaling has not been applied within maximum likelihood estimation. We show an estimation procedure, where the quantifications are chosen so that they maximize the log likelihood of the final model. This was made possible by the employment of the MM algorithm we developed.

We developed a majorization‐minimization algorithm for maximum likelihood estimation of the parameters of the model. We showed that the negative log likelihood for the different response variables can be majorized by a least squares function. For minimizing least squares functions a large body of knowledge is available (cf. Ten Berge, 1993). The algorithm monotonically converges to a minimum. Because each of the original functions per response variable is convex, the sum of these is also convex. Therefore, the attained minimum is also the global minimum. In our application, the algorithm turned out to be quite fast, that is, only a few iterations are needed for convergence. For our cross validation and bootstrap functions we used warm starts to speed up the procedures, where the starting values are equal to the final solutions obtained on the complete data set. We discussed in Section 3.1 that the algorithm might have difficulty in finding the minimum when the estimated variance of the residuals for the numeric response variables is small. In our application and simulation studies, we did not encounter this issue. Other properties of the algorithm still have to be investigated.

For estimation of the model parameters, we make the assumption of conditional or local independence, that is, given the latent variables the responses are independent. This assumption is made in many latent variable models, like item response models, latent class models and some structural equation models. If the assumption is valid, the loss function is a true likelihood function. This allows us to use likelihood based statistics as the AIC and BIC for model selection.

When the local independence assumption is not (completely) valid, this does not need be a problem. The motivation for this statement is as follows. Deen and de Rooij (2020). discuss that for clustered data (e.g. multivariate, longitudinal, repeated measurement data), in order to deal with the within‐individual dependency, the sampling is performed at the individual level rather than at the level of a single measurement of an individual (Davison & Hinkley, 1997). This implies that when a subject is drawn into a specific bootstrap sample, all the observations from this subject are part of that bootstrap sample. The idea behind this is that the resampling procedure should reflect the original sampling procedure (Fox, 2015, p. 662–663). Such clustered resampling has been investigated in several publications, leading to the following conclusions:

- the cluster bootstrap provides consistent estimates of the variances under different models (Field & Welsh, 2007);
- the cluster bootstrap outperforms robust standard errors obtained using a sandwich estimator (GEE) for normally distributed response variables (Harden, 2011; Sherman & le Cessie, 1997);
- the cluster bootstrap yields a consistent approximation of the distribution of the regression estimate, and a consistent approximation of the confidence intervals (Cheng et al., 2013);
- the cluster bootstrap is preferred over linear mixed models or GEE when there are concerns regarding residual covariance structure and distribution assumptions (Feng et al., 1996).

Our cross validation procedure also splits the data based on the individuals, so like in the bootstrap, dependencies in the data are carried over to the training samples in the cross validation procedure. Roberts et al. (2017) show the validity of this approach. Furthermore, when the local independence assumption is not valid, we can view the loss function as a type of quasi‐likelihood. For the analysis of correlated responses, generalized estimating equations (GEE) have been developed that are based on this idea of quasi‐likelhood (Liang & Zeger, 1986; Ziegler et al., 1998). The AIC and BIC statistics have been generalized to this framework and are sometimes called Quasi Information Criteria (QIC; Pan, 2001). In GEE under a working independence assumption the QIC equals the AIC. Yu and de Rooij (2013) investigated such information criteria in the context of longitudinal categorical data also with dimension reduction methods and showed that they possess accurate model selection properties.

In other models for clustered data, specific parameters are included to specifically model the dependencies. The random effects in generalized linear mixed models are an example where a set of subject dependent parameters following a pre‐specfied distribution is included. Another example is latent class models, where the subject specific parameters are categorical. Hubbard et al. (2010) argue that the distributional form of such random variables is not inferable from the data. That is, the data do not provide any information about the form of the distribution and, as such, it remains a guess whether the chosen form is appropriate or not.

In Section 5, we proposed a stepwise model selection procedure in which the rank or dimensionality is first chosen and then a bootstrap procedure is employed to verify which predictor variables have an effect on the responses. Such a stepwise procedure is efficient in terms of the number of models to be fitted. A disadvantage is that the bootstrap does not take into account the uncertainty of the dimension selection procedure. Therefore, the uncertainty estimates from the bootstrap in the second may be to small. Note that if we used, say, likelihood ratio tests for variable election, we would have the same problem. This is a disadvantage of stepwise model selection procedures. Future research should investigate this problem more deeply and verify to what degree the bootstrap confidence regions are affected by the dimension selection in the first step. Furthermore, the stepwise selection of the model does not guarantee to find the best model. Therefore, it would be better to fit models with varying sets of predictors in all possible dimensionalities. Such a procedure requires fitting many models and can be time consuming. Even if we would use the full grid of possible models, different statistics of model selection (AIC and BIC, for example) would probably point towards different best models. In this context, it is also good to point out that we generally do not believe in a true underlying model that we need to recover. Instead, we searched for a model that can describe the data relatively well and is stable.

We applied our method to an empirical data set and compared the results to the results obtained using standard separate regression models for each of the responses. We showed that these standard regression models do not take into account the ordered nature of the predictor variables (as they use dummy variables for the categorical predictors), whereas our model does. When fitting separate models, we need many more parameters than in our reduced rank model, and we showed that this leads to overfitting, that is, large and unstable parameter estimates. In contrast, our GMR^3^ model leads to stable results. Furthermore, our model clearly shows which response variables are affected in a similar way by the predictors and our model can take into account the ordered nature of some of the predictors.

We let the regression weights and the loadings be free parameter estimates. In this sense, the method is exploratory, that is, it finds an underlying structure. In some applications, there might be a priori knowledge about the grouping of response variables or predictor variables. Such knowledge might be incorporated in the future by fixing sets of coefficients to zero, for example, so that a few predictors or responses only pertain to the first or second dimension.

Often, not all predictor variables or response variables are available for all observations, that is, we have missing data. In this paper, we did not consider missing data; we assumed the data to be completely observed. Future work should consider ways to handle missing data within the proposed framework.

For numeric reduced rank regression models, biplot representations have been developed (Ter Braak & Looman, 1994) and also for logistic reduced rank regression models (De Rooij, 2023). Future work might consider biplot representations for our generalized mixed reduced‐rank regression model.

In this paper, we proposed a reduced rank regression model for a mixture of numeric, binary and ordinal response variables. Such response variables are often encountered in the social sciences. In some circumstances, other types of response variables may occur, such as nominal or count variables. We could extend our method to include such response variables. Including nominal response variables should not be too difficult in terms of the algorithm. For a nominal variable, we can assume a multinomial distribution that can be majorized also using quadratic majorization (Groenen & Josse, 2016). However, for interpretation, the reduced rank model of a nominal variable is quite involved. For each category, we would obtain a vector $v_{c}$ of dimension S. The product ${\mathrm{Bv}}_{c}$, does not have a clear interpretation in terms of probabilities or log‐odds. Only the differences of the vectors for two categories obtains a substantial interpretation. For count variables, we would need to develop a majorization function to the Poisson log‐likelihood. Some work on majorization for Poisson variables is done by Landgraf and Lee (2020). It seems that the κ value they use (see Section 3.1 for definition of κ) is the largest observed count. Such a majorization function could lead to the same computational issue as we noted when the estimated residual variance is close to zero. More future research is needed on this topic.

Other future possibilities include the extension of the model to high‐dimensional predictors (i.e. large P) and high‐dimensional responses (i.e. large R). In that case, penalties such as the lasso (Tibshirani, 1996), ridge (Hoerl & Kennard, 1970) or the group lasso (Yuan & Lin, 2006) to penalize the regression weights and/or the loadings. Of course, this requires the development and testing of new algorithms and ways to find optimal penalty parameters. A final future extension, is the generalization of our GMR^3^ method to nested or longitudinal data. We assumed the observations to be independent, but in many applications certain observations might be correlated, for example when observe children in schools, or when we follow participants over time. In such a case, we need to take the dependencies into account, which can be done by including for example random effects as in multilevel models (Kreft & De Leeuw, 1998; Snijders & Bosker, 2011), or by adapting the information criteria (Pan, 2001) and the bootstrap (Deen & de Rooij, 2020; Sherman & le Cessie, 1997). Often, in longitudinal data analysis the interest lies in the development over time for different groups. How to answer such a question when we include optimal scaling of the group variable is something that needs to be investigated.

## AUTHOR CONTRIBUTIONS

**Mark de Rooij:** conceptualization; software; formal analysis; investigation; writing – original draft; writing – review and editing; supervision. **Lorenza Cotugno:** formal analysis; investigation; software; writing – review and editing. **Roberta Siciliano:** conceptualization; supervision; writing – original draft; writing – review and editing.

## FUNDING INFORMATION

The first author (Mark de Rooij) is part of the GUTS program which is funded by an NWO Gravitation programme supported by the Dutch Ministry of Education, Culture and Science of the government of the Netherlands, Grant nr 024.005.011. Furthermore, he was a fellow at the Netherlands Institute for Advanced Studies (NIAS) in Amsterdam when working on the revisions of the manuscript. R. Siciliano and L. Cotugno were supported by the Italian Ministry of Research, under the complementary actions to the NRRP “Fit4MedRob ‐ Fit for Medical Robotics”, Grant number PNC0000007).

## CONFLICT OF INTEREST STATEMENT

The authors have no relevant financial or non‐financial interests to disclose.

## Data Availability

The data that support the findings of this study are available in eurobarometer at https://europa.eu/eurobarometer/screen/home. These data were derived from the following resources available in the public domain: – Eurobarometer, https://europa.eu/eurobarometer/screen/home. – Github, https://github.com/mjderooij/Generalized‐Mixed‐Reduced‐Rank‐Regression. The Eurobarometer data are freely available from the the website of the European Union https://europa.eu/eurobarometer/screen/home. R‐code for the analyses of the data set is available on the github‐page of the first author (https://github.com/mjderooij/Generalized‐Mixed‐Reduced‐Rank‐Regression). Also the R‐code for the simulation study can be found on that github page.

## Footnotes

^1^
The number of parameters to estimate depends also on the number of response variables but we will not select the response variables, all are included.

^2^
Note that negative McFadden's adjusted R‐squared is possible.

^3^
The idea behind BIC is that the maximum likelihood estimators for arbitrary nowhere vanishing a priori distributions can be obtained as large‐sample limits of the Bayes estimators. Thus, a suitable modification of maximum likelihood is searched, through the analysis of the asymptotic behavior of Bayes estimators under a special, not absolutely continuous, class of priors.

## APPENDIX A COMPARISON TO SEPARATE MODELS

We fitted standard binary and ordinal logistic regression models to the seven response variables. As predictors, we used age, political alignment, gender, degree of urbanization and education level. The categorical predictor variables were coded using dummies, with the first category as the baseline. As, all predictors were of importance in our gmr3 model, we did not do any further model selection. The sum of deviances of these seven models is 9923.01 (corresponding to ℒ of 4961.51). Of course, this value is lower compared to our analysis, because more parameters are fitted. That is, the total number of parameters is 115. The corresponding AIC equals 10153.01, while the BIC is 10696.94. Both values are higher than those for our selected model.

The parameter estimates obtained using the seven separate models are given in Table A1.

**TABLE A1:** Coefficients of separately fitted logistic or ordinal logistic regression models.

	T	FE	CI	MW	FS	DI	RE
A	−.18	−.32	−.04	.09	.16	.46	.25
PA2	−.96	−.36	−.81	−.59	−.56	.17	−.59
PA3	−1.30	−.98	−1.40	−.99	−.72	.35	−1.04
G2	−.18	.02	−.22	.02	−.40	−.53	−.21
U2	.06	−.01	.03	−.04	.11	−.00	.02
U3	.69	.15	.37	.18	.40	−.07	.41
E2	−.71	.12	−1.54	−10.14	−13.26	−12.48	−11.87
E3	−.35	.33	−.99	−11.19	−12.36	−12.99	−11.88
E4	−.26	.04	−.98	−10.92	−12.03	−13.22	−12.14
E5	−.13	2.24	−.05	−11.02	−12.05	−12.51	−11.27
E6	.22	−.07	−.71	−11.08	−12.21	−13.19	−11.85
E7	.09	.14	−.21	−11.19	−12.00	−13.46	−11.93
E8	.65	.14	−.18	−11.43	−11.71	−13.18	−12.04
E9	1.52	.87	.51	−10.14	−10.04	−13.02	−12.55

To compare the fitted parameters of the separate models with those of our model, we need to compute some implied coefficients. The regression coefficients corresponding to the dummy variables specify a difference in the log odds between two categories of a predictor variable. We can obtain similar coefficients from our model. We already saw the implied coefficients ${\mathrm{BV}}^{\prime }$. To obtain the effect of two predictor categories, we need to incorporate the optimal scaling. Suppose, we like to know the effect of the second category against the first of the first predictor on the different response variables. Then we need to compute $(\phi_{1}(2)-\phi_{1}(1))b_{1}^{\prime }V^{\prime }$. Here $b_{1}$ is the S‐dimensional vector of coefficients for the first predictor, and $\phi_{1}(1)$ is the optimally scaled value for category 1 of the first predictor variable and $\phi_{1}(2)$ the optimally scaled value for category 2 of the first predictor variable. Similarly, for the third against the first category of the first predictor we need $(\phi_{1}(3)-\phi_{1}(1))b_{1}^{\prime }V^{\prime }$. In this way, we can compare the effects obtained in the separate models to the effects obtained in our model. The implied coefficients are given in Table A2

**TABLE A2:** Implied coefficients of GMR3 model.

	T	FE	CI	MW	FS	DI	RE
A	−.16	−.27	−.05	.06	.17	.50	.23
PA2	−.96	−.42	−.90	−.33	−.57	.16	−.46
PA3	−1.55	−.68	−1.45	−.53	−.92	.26	−.75
G2	−.12	.13	−.21	−.14	−.31	−.41	−.34
U2	.04	.02	.04	.02	.03	−.00	.02
U3	.53	.20	.51	.20	.35	−.02	.30
E2	.00	.00	.00	.00	.00	−.00	.00
E3	.28	.13	.25	.09	.15	−.08	.11
E4	.33	.16	.30	.11	.18	−.09	.14
E5	.64	.31	.58	.20	.34	−.18	.26
E6	.64	.31	.58	.20	.34	−.18	.26
E7	.81	.39	.74	.26	.43	−.23	.33
E8	.97	.47	.89	.31	.51	−.27	.40
E9	1.77	.86	1.62	.56	.94	−.49	.73

Whereas some of the coefficients of the separate models are really large, we see that all our coefficients behave well. Because, we assumed some predictor variables to be ordinal, we see that the implied coefficients are neatly ordered, whereas such ordering is absent from the fitted separate models.

We can also look at the standard errors of these coefficients. Applying the bootstrap, and computing these coefficients in each of the bootstrap samples, we can obtain a bootstrap standard error by computing the standard deviation over the bootstrap samples. For the separately fitted models these standard errors are given in Table A3. The corresponding standard errors of our model are given in Table A4. It can be verified that the results of our model are much more stable, that is, the standard errors are much smaller overall. In other words, the efficiency of the our estimator is much higher, specifically for the categorical predictors.

**TABLE A3:** Bootstrap standard error estimates of separately fitted models.

	T	FE	CI	MW	FS	DI	RE
A	.08	.08	.07	.08	.08	.07	.07
PA2	.20	.18	.16	.19	.20	.16	.16
PA3	.22	.21	.21	.21	.24	.19	.20
G2	.17	.16	.15	.17	.18	.15	.15
U2	.18	.17	.16	.18	.19	.16	.17
U3	.22	.20	.19	.21	.24	.20	.21
E2	11.17	10.79	.84	4.76	3.62	4.61	4.47
E3	10.08	9.96	.42	3.22	3.55	4.27	4.37
E4	10.09	9.97	.40	3.22	3.55	4.27	4.36
E5	10.86	11.88	.87	5.25	6.18	6.16	6.35
E6	10.10	9.99	.45	3.24	3.58	4.29	4.36
E7	10.09	9.98	.40	3.23	3.55	4.28	4.38
E8	10.10	9.98	.40	3.22	3.55	4.27	4.36
E9	10.71	10.08	.52	2.56	5.34	.86	.99

**TABLE A4:** Bootstrap standard error estimates of GMR3 models.

	T	FE	CI	MW	FS	DI	RE
A	.09	.08	.07	.10	.08	.08	.09
PA2	.16	.10	.13	.13	.13	.12	.14
PA3	.20	.17	.19	.20	.17	.20	.21
G2	.14	.11	.13	.10	.14	.15	.11
U2	.10	.04	.09	.04	.07	.04	.06
U3	.17	.11	.15	.11	.15	.16	.15
E2	.37	.21	.31	.09	.16	.22	.12
E3	.52	.30	.43	.13	.24	.32	.17
E4	.54	.32	.45	.14	.26	.35	.20
E5	.54	.33	.45	.15	.26	.38	.21
E6	.54	.33	.45	.16	.27	.39	.22
E7	.54	.35	.45	.17	.28	.44	.24
E8	.64	.39	.53	.22	.37	.50	.31
E9	.70	.48	.57	.31	.46	.64	.40
